# The silent half

**DOI:** 10.1016/j.lanwpc.2021.100137

**Published:** 2021-03-25

In September, 1995, the Beijing Declaration and Platform for Action was announced at the Fourth World Conference on Women in Beijing, China. The framework, covering 12 key areas in which swift action is needed to achieve greater gender equality, is recognised as a landmark resolution advocating for women's rights. Remarkable progress on economic empowerment and educational attainment has been made since then, resulting in the improved health and wellbeing of women, as exemplified by halving of the maternity mortality rate since 2000 in the Asia-Pacific region. Yet much more work remains to be done.

Statistically, women live longer than men, but they also face more crises across their lifespan. Data from the International Labour Organization shows that women in the Asia-Pacific region do around 80% of unpaid care work. It was estimated that 20% of women in the region have experienced lifetime intimate partner violence, and that proportion reaches almost 64% in Fiji and the Solomon Islands. Gender norms and stigma for some diseases and health conditions prevent women from seeking health care and treatment. We need to create tailored approaches to deliver health-care services to women who are disadvantaged in socioeconomic and health status because the health-care systems fail them time and again. In the COVID-19 pandemic, women have encountered increased gender-based violence and a heavier burden of unpaid care and work, while their incomes have shrunk due to job loss. Deprioritisation in basic health care such as sexual and reproductive health services squeezes medical resources available for women, which could trigger a secondary pandemic of women's health. It is estimated that there is a rising rate of unintended pregnancies, maternal mortality and morbidity, and sexually transmitted diseases in low-resource areas since last year.

To reduce existing heath inequality, specifically within health systems, a voice from women is needed. Women's leadership has positively contributed to effective pandemic control in many countries, such as New Zealand. Women make up a large proportion (90%) of frontline health-care workers in China. However, according to the latest Global Health 50/50 report, men take up 70% of senior positions in the 201 surveyed medical organisations. For this year's International Women's Day, the UN calls for Women in Leadership to achieve gender equality in the post-pandemic era. This aim is particularly important for the Western Pacific region, where women and girls are more likely to take professions with lower occupational prestige and perceived skill level, while social norms and gender stereotypes in many Asian countries prevent them from taking a more senior challenging job.

To pave the way towards empowering women with decision making positions and to improve gender disparities in health, more gender-disaggregated data are needed. Such data can bring forth and amplify the silent suffering that women are enduring, with the hope to develop better policies and programmes and enable effective changes in different governmental sectors. Gender-based research on health is largely led by high-income countries, and more data from low-income and middle-income countries are welcomed to highlight specific needs from women in resource-limited areas. In this issue of *The Lancet Regional Health – Western* Pacific, valuable data from Islay Mactaggart and colleagues reveal the shortage of basic water, sanitation, and hygiene (WASH) facilities among menstruating women and people with disabilities in northern Vanuatu. Such an evidence-based study urges the government and relevant organisations to put gender and disability-inclusive WASH policies in place for those left-behind groups to meet their WASH requirements in the future.

The consequences of the pandemic for women's health will be long-lasting. However, the pandemic offers an opportunity for us to rethink existing gender inequality in health and to find a way to change, to build a post-pandemic era when the different health needs of women are recognised and met. The ultimate aim is to achieve a world with greater gender equality, as advocated for in the *Beijing Declaration and Platform for Action.*

*The Lancet Regional Health – Western Pacific*

